# Buyang Huanwu Decoction Promotes Angiogenesis after Cerebral Ischemia by Inhibiting the Nox4/ROS Pathway

**DOI:** 10.1155/2020/5264205

**Published:** 2020-07-31

**Authors:** Jian Shen, Kaiyuan Huang, Yu Zhu, Kangli Xu, Renya Zhan, Jianwei Pan

**Affiliations:** Departments of Neurosurgery, First Affiliated Hospital, School of Medicine, Zhejiang University, Hangzhou, Zhejiang Province, China

## Abstract

**Background:**

Buyang Huanwu decoction (BYHWD), an important traditional Chinese medicine (TCM), has been used clinically for centuries for the treatment of various diseases. The study aims to explore the BYHWD effects on angiogenesis and neuroprotection after cerebral ischemia/reperfusion (CI/R) injury in rats and to explore the underlying angiogenic roles and mechanisms of BYHWD in hydrogen peroxide (H_2_O_2_) induced oxidative stress in human umbilical vein endothelial cells (HUVECs) model.

**Methods:**

The effects of BYHWD on neurological function were screened by measuring neurological deficits, spatial memory function, and angiogenesis (by microvascular density (MVD) and cerebral blood flow (CBF)) after CI/R injury in middle cerebral artery occlusion (MCAO) in vivo in rats. In vitro, we examined the angiogenic roles and mechanisms of action of BYHWD in an H_2_O_2_-induced oxidative stress HUVECs model by measuring cell viability, apoptosis, vascular tube formation, intracellular ROS generation, NADPH oxidase (Nox) activity, and Nox4 protein expression.

**Results:**

BYHWD significantly improved neurological function, including neurological deficits and spatial learning and memory, and significantly increased MVD and CBF in the ischemic penumbra after CI/R injury in rats. BYHWD significantly increased cell viability, inhibited apoptosis, induced vascular tube formation, decreased intracellular ROS generation, and reduced Nox activity and Nox4 protein expression in H_2_O_2_-treated HUVECs in a dose-dependent manner.

**Conclusions:**

Our study demonstrates that BYHWD promotes neurological function recovery and increases angiogenesis. BYHWD exerts angiogenic effects against cerebral ischemic injury through the downregulation of Nox4, which results in the reduction of ROS generation.

## 1. Introduction

Cerebral ischemic stroke remains the main cause of morbidity and mortality in adults worldwide. An epidemiological survey of stroke in China showed that the incidence, prevalence, and mortality rates of stroke were 246.8, 1,114.8/100,000 population and 114.8/100,000 person-years, respectively [1]. Angiogenesis is considered an important neurovascular response for the repair of a stroke-damaged brain [2]. A higher density of the new blood capillaries is associated with lower mortality and better neurological outcome in ischemic stroke patients [2], suggesting that enhancing active angiogenesis in the ischemic area may be an effective new approach for stroke recovery. However, too much reactive oxygen species (ROS), e.g., hydrogen peroxide (H_2_O_2_), hydroxyl radicals, and superoxide radicals after cerebral ischemia/reperfusion (CI/R) are the main mechanisms contributing to neurological toxicity and blood-brain-barrier (BBB) disruption, leading to oxidative damage and cell death [3–5]. Previous studies have indicated that secondary brain injury is aggravated by ROS-induced apoptosis of vascular endothelial cells (VECs) after cerebral ischemic stroke [6, 7]. Therefore, protecting VECs against ROS-induced injury might have a therapeutic benefit for ischemic stroke.

Many studies have focused on inhibiting the generation of ROS, especially a ROS-induced ROS release mechanism that has been demonstrated by several recent studies [5, 8]. Endothelial cells produce a large number of ROS that reduce sensitivity to exogenous ROS and antioxidant activity in CI/R injury [9]. Similarly, ROS are produced by neurons increasing endothelial cells damage by oxygen free radicals [10]. ROS are produced from different sources in the body, e.g., the mitochondrial electron transport chain, xanthine oxidase (XO), NADPH oxidases uncoupled nitric oxide synthase (NOS) myeloperoxidase, and cytochrome p450. NADPH oxidase is regarded as a main source of ROS in endothelial cells, in which Nox1, 2, 4, and Nox5 are expressed [11]. Of these, Nox4 belongs to the ROS-generating NADPH oxidase family involved in endothelial cell angiogenesis [12, 13], which makes it a relevant target for angiogenesis therapy after CI/R injury. Buyang Huanwu decoction (BYHWD) is a famous traditional Chinese medicine (TCM) formula that has been clinically used for the prevention and treatment of cerebrovascular accidents in China for centuries. Several clinical trial studies have shown that BYHWD improves the prognosis of ischemic stroke [14]. Clinical and preclinical studies indicate that BYHWD is generally safe, improves neurological deficits in patients with acute cerebral ischemic stroke, and confers neuroprotection in experimental stroke models [15, 16]. BYHWD stimulates adult neurogenesis and angiogenesis processes after cerebral ischemic injury [7, 17, 18]. Therefore, angiogenesis may be directly associated with neurogenesis after cerebral ischemic stroke. However, the mechanisms underlying these effects remain unclear. Based on these findings, we aimed to investigate the neuroprotective and angiogenesis functions of BYHWD through the Nox4/ROS pathway.

## 2. Materials and Methods

### 2.1. BYHWD Preparation and Quality Control

A previously published protocol was adopted for BYHWD preparation [19]. In brief, Milkvetch (*Radix Astragali*), Chishao (*Radix Paeoniae Rubra*), Taoren (*Semen Persicae*), Safflower (*Flos Carthami*), Danggui (*Radix Angelicae Sinensis*), and Rhizoma Ligustici Chuanxiongand Lumbricus (*Pheretima aspergillum*) were thoroughly mixed in a ratio of 120, 10, 10, 10, 10, 10, and 4.5. The decoction was prepared through boiling this mixture for 30 minutes at 100°C in 10 times the volume of distilled water. Then, the drug-containing solution was removed and the residue was boiled again twice. The drug-containing solution was mixed and dried to a powder. Quality was checked by high-performance liquid chromatography (HPLC) by a method adapted from our previous study [7].

### 2.2. In Vivo Studies

#### 2.2.1. Animals, Grouping, and Administration of the Drug

Male SD rats having body weight ranging from 220–250 g were acquired from the Animal Sciences Laboratory, Academy of Medical Sciences, Zhejiang (China), and divided into four groups randomly, i.e., vehicle group (middle cerebral artery occlusion (MCAO)), sham group, low-dose group (MCAO treated with BYHWD 12.5 g/kg per day), and high-dose group (MCAO treated with BYHWD 25 g/kg per day). The drug was administered orally twice a day. The sham and vehicle groups were administered distilled water. The experimental protocols were approved by the Committee for Animals Research Ethics, School of Medicine, Zhejiang University, China.

#### 2.2.2. Induction of Focal CI/R Injury Model

Focal cerebral ischemia was induced by MCAO in rat models following a previously published method [17]. In brief, using 3% isoflurane, rats were anesthetized and were placed on the heating bed to maintain their rectal temperature at 37°C. Then, from the external cerebral artery (ECA) stump, a silicone coated 4-0 monofilament suture was inserted and advanced slightly to the middle cerebral artery (MCA) for occlusion. The suture was removed following 90 min of occlusion.

#### 2.2.3. Assessment of Neurological Deficit

Bederson et al.'s method [20] was used to assess the neurological deficit score once a week. Three examiners who remained blinded to the experimental protocol were employed for scoring each rat. In brief, the neurological deficit was classified as 0, 1, 2, 3, and 4 (whereas no noticeable deficit = 0, fore-limb flexion = 1, fore-limb flexion along with reduced resistance to lateral push = 2, unidirectional circling = 3, and unidirectional circling with decreased consciousness = 4). If the animals were with no deficit after surgery, they were removed from the research.

#### 2.2.4. Morris Water Maze (MWM) Test

Spatial memory assessment in animals was done through the MWM test as described previously [21]. Spatial learning tests were started 4 weeks after surgery and consisted of providing a block of trials twice daily for five consecutive days where rats were required to locate the platform when it was submerged below (1 cm) the water surface. The rats were placed into the water in a randomized manner at each of the four possible locations of the apparatus. Each test lasted until 120 seconds or till the rat climbed onto the platform. The animals that failed to find the platform within 120 seconds were manually guided to the platform. All rats were stayed for 30 seconds on the platform, and then after 30 seconds, the platform was removed, and the number of times the rats crossed the platform position during a 3 min session was recorded using a video analysis system.

#### 2.2.5. Immunofluorescence Staining

Using isoflurane (3%), the animals were anesthetized and perfused with normal saline transcardially followed by paraformaldehyde (4%). The brains of animals were fixed for 8 h in paraformaldehyde (4%) for 8 h, cryoprotected in 10%, 15%, and 20% sucrose for 1 d each, and frozen in liquid nitrogen. Frozen sections were prepared for immunofluorescence staining. Coronal sections having 20 *μ*m thickness were obtained using a cryostat (Microm HM560, Germany). The tissue slices were fixed for 10 min in acetone, treated for 2 h with 10% goat serum, and incubated with primary rabbit anti-factor VIII (1 : 200; Santa Cruz, USA), followed with goat anti-rabbit antibody conjugated with tetramethylrhodamine (TRITC; 1 : 200; Jackson Lab, USA) for 2 h. This was followed by their incubation with 4,6-diamidino-2-phenylindole (DAPI) for counterstaining of their nuclear DNA. Tissue slices were studied with a laser-scanning confocal microscope and evaluated with Image Pro-Plus software (Version 6, Media Cybernetics).

#### 2.2.6. Assessment of Cerebral Blood Flow (CBF)

Laser-Doppler cerebral blood flow detector was used for the measurement of local CBF as previously described [22]. In brief, experimental animals were anesthetized using 3% isoflurane, and the scalp was incised to expose the brain above the right MCA region. The laser was fixed for at least 5 minutes on the ischemic location (5 mm lateral and 1.5 mm posterior to bregma) and the average value was noted. Data were evaluated with LDPI Win 2 software (Perimed AB, Sweden).

### 2.3. In Vitro Experiments

#### 2.3.1. Cell Culture and Preparation of BYHWD Extraction

HUVECs were acquired from ATCC (Rockville, USA) and maintained in DMEM supplemented with heat-inactivated fetal bovine serum (FBS; 10%) in a CO_2_ (5%) humidified temperature at 37°C. After the preparation of BYHWD extraction as described above, it was centrifuged for twenty min at 3000 ×*g*, and the cell supernatant was collected and then filtered using a 0.22 *μ*m microporous filter membrane and stored at −20°C for future experimentation. The BYHWD was diluted to different concentrations in the medium before use.

#### 2.3.2. Cell Viability Assay

Cell viability was determined through 3-(4,5-dimethylthiazol-2-yl)-2,5-diphenyl tetrazolium bromide (MTT, Sigma, USA) procedure as described by the previously published report [23]. The cells were divided into control, H_2_O_2_, and H_2_O_2_ + BYHWD (10, 20, and 30 mg/ml) groups. HUVECs were seeded at a density of 2 × 10^3^ cells/well into 96-well plates (Thermo Scientific), followed by incubation for 12 h in serum-free medium, then pretreated for 6 h with different BYHWD concentrations (10, 20, or 30 mg/ml), and exposed to H_2_O_2_ (400 *μ*M) for 4 h and 6 h. Cells were incubated for 3 h at 37°C with 5 mg/ml MTT solution (150 *μ*L). MTT mixture was removed and cells were incubated with formazan crystals dissolved in dimethyl sulfoxide (DMSO, 100 *μ*L). The optical density (OD) was determined at 490 nm with enzyme-labeled Biotek ELX 800 instrument (Winooski, VT, USA).

#### 2.3.3. Apoptosis Assay

Apoptotic cells were tested using Hoechst 33342 and propidium iodide (PI) (Hangzhou Baoke Biological Technology Co. Ltd., Hangzhou, Zhejiang, China) staining assay as described previously [24]. The cells were divided into control, H_2_O_2_, BYHWD (30 mg/ml), and H_2_O_2_ + BYHWD (10, 20, or 30 mg/ml) groups. After incubation, the cells were stained twice at 37°C for ten min with Hoechst 33342, and then, PI and data were expressed as a percentage of apoptotic cells with Image Pro-Plus software (V.6.0, Rockville, USA).

#### 2.3.4. Vascular Tube Formation

Vascular tube formation was measured as an indicator of angiogenesis in HUVECs. HUVECs were pretreated with various concentrations of BYHWD (10, 20, or 30 mg/mL) for 6 h and then exposed to H_2_O_2_ (400 *μ*M) for 8 h and 10 h. The tubular formation of endothelial cells was observed under a microscope, and the number of vascular structures was determined with Image Pro-Plus software (V.6.0Media, Rockville, USA).

#### 2.3.5. Estimation of Intracellular ROS

Intracellular ROS was measured based on ROS-induced conversion of nonfluorescent 2′,7′-dichlorofluorescin diacetate (2′,7′-DCFH-DA) to fluorescent DCFH [25]. The cells were divided into control, H_2_O_2_ (400 *μ*M), BYHWD (30 mg/ml), H_2_O_2_ + BYHWD (10, 20, or 30 mg/ml), and H_2_O_2_ + apocynin (200 *μ*M) groups. Apocynin is an NADPH oxidase inhibitor that inhibits the activity of Nox4 and decreases the generation of ROS in cells [3] and was used as a positive control. HUVECs were cultured into BD Falcon® 6-well plates (BD Biosciences, USA), pretreated with various known concentrations of BYHWD (i.e., 10, 20, or 30 mg/ml) for 6 h and apocynin for 1h, and then exposed for 6 h to H_2_O_2_ (400 *μ*M). After incubation as described above, the cells were three times washed before incubation in 50 *μ*M DCFH-DA solution at 37°C for 3 h. The DCFH fluorescence was excited at 485 nm and emitted at 520 nm and was examined under a fluorescent microscope (Olympus, Tokyo, Japan).

#### 2.3.6. NADPH Oxidase Assay

NADPH oxidase activity was assessed by the previously reported lucigenin chemiluminescence method using commercial kits (Jiancheng Biological Materials Co. Nanjing, China) [26]. The cells were divided into control, H_2_O_2_ (400 *μ*M), BYHWD (30 mg/ml), apocynin (200 *μ*M), H_2_O_2_ + BYHWD (10, 20 or 30 mg/ml), and H_2_O_2_ + apocynin (200 *μ*M) groups. HUVECs were pretreated with different known concentrations of BYHWD (i.e., 10, 20, or 30 mg/ml) for 6 h and apocynin for 1 h and then contacted with H_2_O_2_ (400 *μ*M) for 2 h in the diluted culture medium. After incubation, cells were lysed and centrifuged at 4000 ×g at 4°C for 5 min at 4°C, then 100 *μ*L of PBS (to which protease inhibitor was added in advance) was added to freeze-thaw (−80°C–37°C water) the cells 4 times and then centrifuged (12000 ×*g* at 4°C for 10 min) to obtain the protein solution. A phosphate buffer having 1 mmol/L EGTA, 5 pmol/L lucigenin (electron acceptor), 150 mmol/L sucrose, and 100 *μ*M NADPH (reaction substrate) was added, and then 50 *μ*L protein solution was added to each well to start the reaction. NADPH oxidase activity was expressed as relative chemiluminescence units/second/mg of protein.

#### 2.3.7. Western Blotting

To determine the time-dependent effect of H_2_O_2_ on Nox4 protein expression in HUVECs, the cells were treated with H_2_O_2_ (400 *μ*M) for different time intervals (30 min, 1, 2, or 4 h). To determine the BYHWD effects on H_2_O_2_-induced Nox4 protein expression, the cells were divided into control, H_2_O_2_ (400 *μ*M), BYHWD (30 mg/ml), and H_2_O_2_ + BYHWD (10, 20 and 30 mg/ml) groups. The cells were pretreated with various known concentrations of BYHWD (i.e., 10, 20, or 30 mg/ml) for 6 h. Bicinchoninic acid (BCA) protein assay kits (Sigma, China) were used for the quantification of protein extracts. Proteins were separated in equal amounts (50 *μ*g per lane) using gel electrophoresis technique on SDS-polyacrylamide (10%) gel and transferred to polyvinylidene difluoride Millipore membrane (Thermo Scientific, USA). Membranes were incubated overnight at 4°C in a ratio of 1 : 1000with goat anti-glyceraldehyde-3-phosphate dehydrogenase (GAPDH; Sigma) or primary goat anti-Nox4 (Abcam, USA) followed by incubation for 3 h at room temperature with secondary rabbit antibody conjugated to HRP (1 : 3000, Cell Signaling, USA). Then, employing higher chemiluminescence and X-ray film (Kodak, USA), the signals were visualized and data were presented as the ratio of Nox4 to GAPDH.

### 2.4. Statistical Analyses

All results were presented as mean ± standard deviation (SD). SPSS software (version 19.0, IBM, USA) was used for statistical analyses. The analysis of variance and Student's *t*-test were applied for statistical analyses, and statistical significance was defined as *P* < 0.05.

## 3. Results

### 3.1. Effect of BYHWD on Neurological Deficits after Focal Cerebral Ischemia

**Figure 1:**
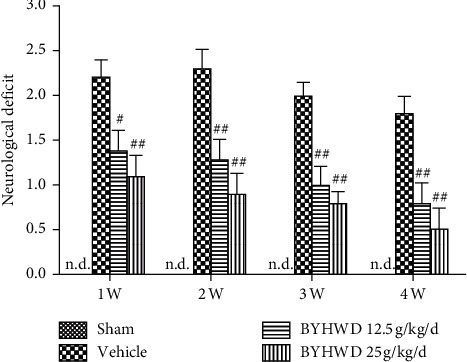
Effects of BYHWD on neurological deficit after focal cerebral ischemia in rats. The neurological deficit for each group was measured at 1, 2, 3, and 4 w after focal cerebral ischemia. ^#^*P* < 0.05 and ^##^*P* < 0.01 versus vehicle group on the same day (*n* = 10).

Bederson's score was used for assessing neurological function and evaluation of neurological deficits after MCAO. After 1–4 weeks of administration of BYHWD, the neurological deficit in both low- and high-dose treatment groups was statistically lower (*P* < 0.01) than that of vehicle-treated rats, and the neurological deficit decreased gradually during the 4 w of administration of BYHWD (Figure 1). The results showed that BYHWD improved the neurological deficit after focal cerebral ischemia in a dose-dependent manner.

### 3.2. Effects of BYHWD on Spatial Acquisition after Focal CI

**Figure 2:**
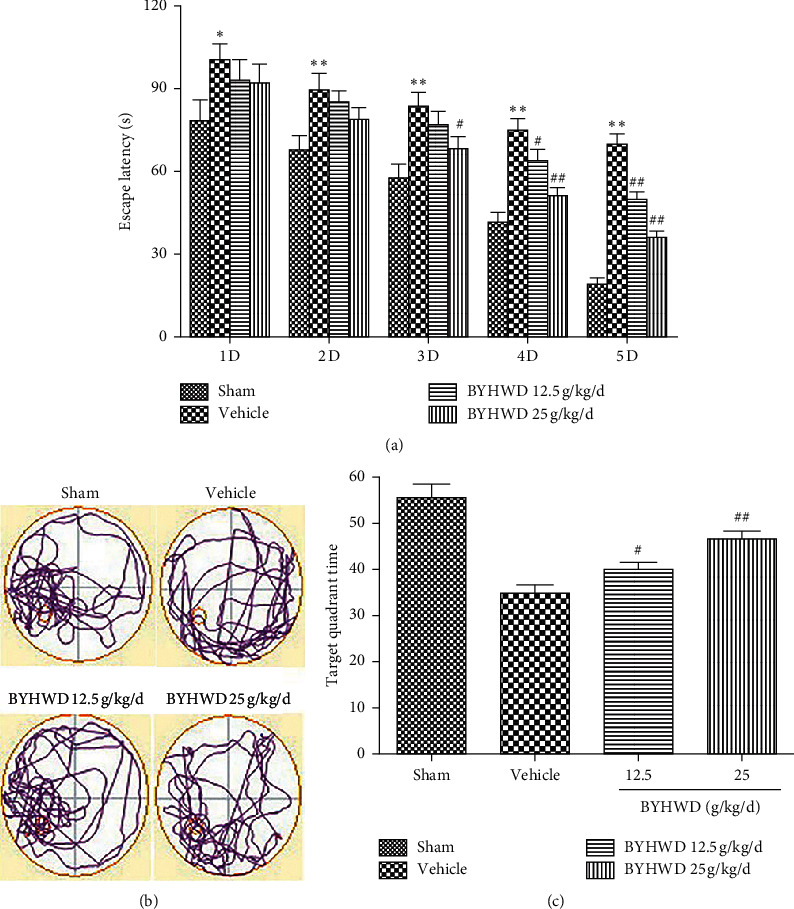
Effects of BYHWD in spatial acquisition trials and probe trials of Morris water maze after focal cerebral ischemia in rats. (a) Latency for reaching the hidden platform during 5 consecutive days of spatial acquisition training. (b) Representative search patterns during the probe transfer trial. (c) Time spent in the quadrant where the platform had previously been located during the probe transfer trial. ^#^*P* < 0.05 and ^##^*P* < 0.01 versus vehicle group on the same day (*n* = 10).

The MWM experiment was carried out to evaluate spatial learning and memory function. The time taken to find the platform became gradually shorter in all groups during the training of the spatial memory task. From the first to the fifth day of training, the time to find the platform in the sham group was statistically less compared to the vehicle group (*P* < 0.05 or *P* < 0.01). On the third day of training, the duration of finding the platform was significantly lower in the high-dose BYHWD treatment group compared to the vehicle group (*P* < 0.05). On the fourth and fifth days of training, the time to find the platform was significantly less in both the low- and high-dose treatment groups, compared with that of the vehicle group (*P* < 0.05 or *P* < 0.01; Figure 2(a)). After 5 d of training, the duration spent in the previously located platform in a quadrant was significantly lengthier in the BYHWD treatment group (*P* < 0.05 or *P* < 0.01; Figure 2(b)). The data indicate that BYHWD improved memory function and spatial learning after cerebral ischemia in a dose-dependent manner (Figure 2(b)).

### 3.3. Effects of BYHWD on MVD after Focal CI

**Figure 3:**
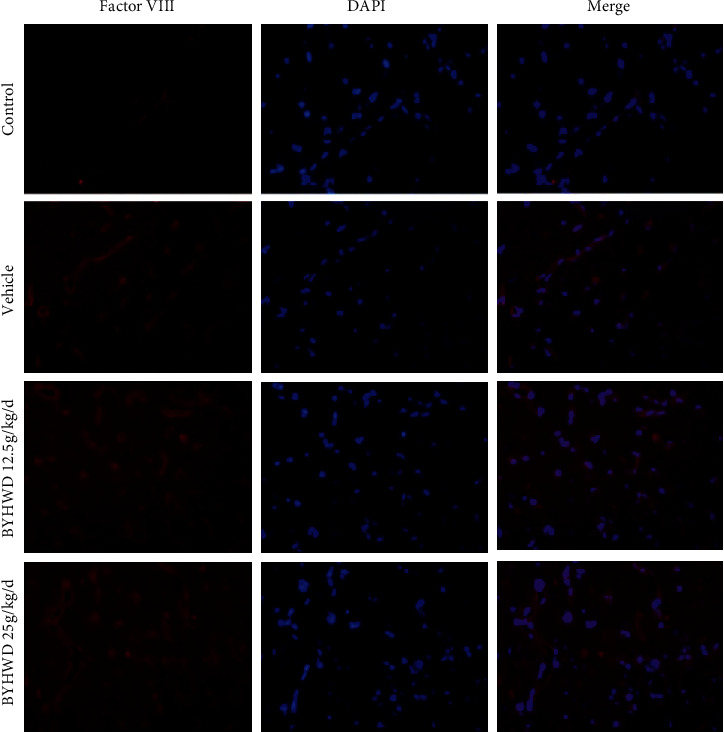
Effects of BYHWD on microvessel density (MVD) after focal cerebral ischemia in rats. Representative microphotographs of factor VIII-positive MVD for each group at 4 w after focal cerebral ischemia (*n* = 6). Scale bar, 50 *μ*m.

Immunofluorescence staining for the endothelial cell-specific antigen factor VIII was used for the evaluation of microvessel density (MVD) in the ischemic penumbra [27]. The MVD was low in the sham group as compared to the vehicle group at 4 w after cerebral ischemia. MVD was significantly higher after 4 w of administration of BYHWD, compared to the vehicle group (Figure 3). These data demonstrate that BYHWD improved the angiogenesis of the ischemic area after focal cerebral ischemia.

### 3.4. Effects of BYHWD on Cerebral Blood Flow (CBF) after Focal CI

**Figure 4:**
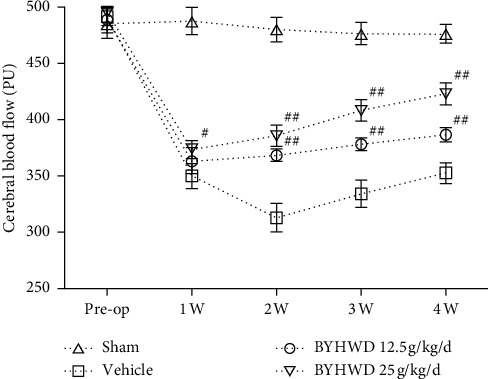
Effect of BYHWD on cerebral blood flow (CBF) after focal cerebral ischemia in rats. CBF for each group before the operation, and 1, 2, 3, and 4 w after focal cerebral ischemia. ^#^*P* < 0.05 and ^##^*P* < 0.01 versus vehicle group on the same day (*n* = 10).

CBF in the ischemic area was assessed to measure neurovascular remodeling and restoration of functional blood supply. CBF in the ischemic area increased gradually from 1 to 4 weeks after administration of BYHWD. CBF was statistically higher compared with the vehicle group in the high-dose treatment group only at 1 w (*P* < 0.05), while at 2–4 w, CBF was significantly higher in both low- and high-dose treatment groups (*P* < 0.01; Figure 4). The results indicate that BYHWD improves the CBF in the ischemic area after cerebral infarction in a dose-dependent manner.

### 3.5. Effect of BYHWD on H_2_O_2_-Induced Cytotoxicity

**Figure 5:**
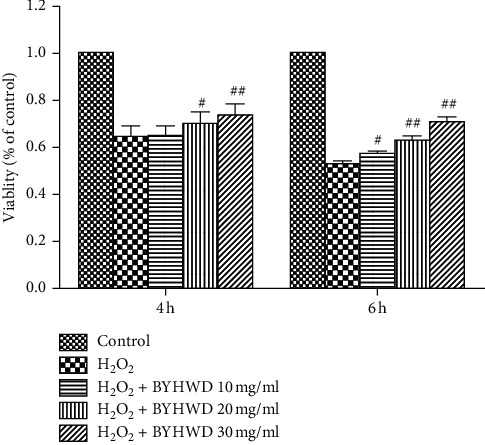
Effects of BYHWD on H_2_O_2_-induced cytotoxicity in HUVECs. Cell viability in HUVECs preincubated with BYHWD (10, 20, or 30 mg/ml) for 6 h followed by H_2_O_2_ for 4 or 6 h ^#^*P* < 0.05 and ^##^*P* < 0.01 versus H_2_O_2_ group at the same time.

MTT assay was used for measuring the viability of BYHWD-treated HUVECs. The cell viability was significantly higher in cells treated with BYHWD (20 & 30 mg/ml; *P* < 0.05 or *P* < 0.01). After 4 h treatment with H_2_O_2_, cell viability was statistically higher in the 20 and 30 mg/ml BYHWD treatment groups (*P* < 0.01), compared with the H_2_O_2_ group. After 6 h treatment with H_2_O_2_, cell viability in cells treated with 10, 20, or 30 mg/ml BYHWD was significantly higher, and the effect was dose-dependent (Figure 5). These data recommend that BYHWD shields HUVECs from H_2_O_2_-induced cytotoxicity.

### 3.6. Effects of BYHWD on H_2_O_2_-Induced Cell Apoptosis in HUVECs

**Figure 6:**
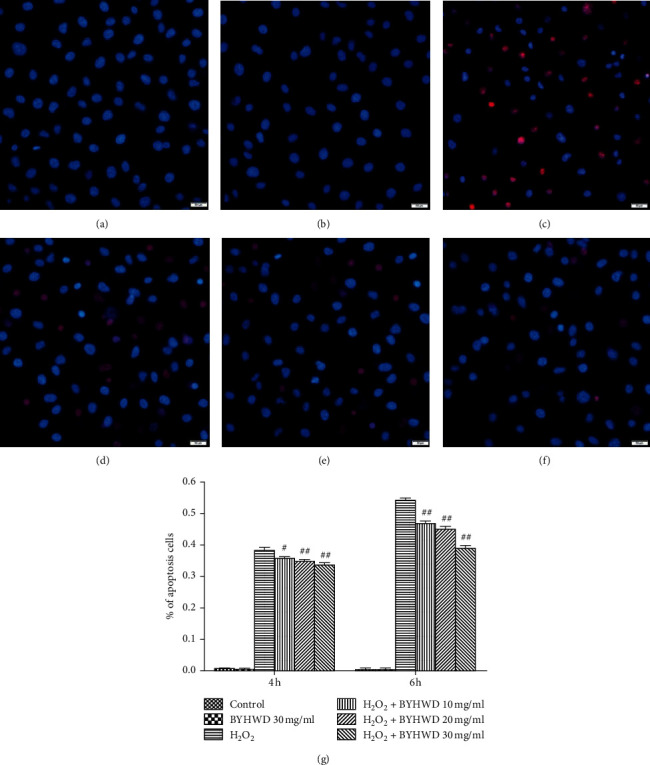
Effects of BYHWD on H_2_O_2_-induced cell apoptosis in HUVECs. Cells were double-stained with cell membrane-permeable (Hoechst 33342; blue) and impermeable (PI; red) DNA labeling fluorochromes. (a) Vehicle medium (control) for 6 h. (b) Preincubation with BYHWD (30 mg/ml) for 6 h. (c) H_2_O_2_ (400 *μ*M) for 6 h. (d) Preincubation with BYHWD (10 mg/ml) for 6 h followed by H_2_O_2_ for 6 h. (e) Preincubation with BYHWD (20 mg/ml) for 6 h followed by H_2_O_2_ for 6 h. (f) Preincubation with BYHWD (30 mg/ml) for 6 h followed by H_2_O_2_ for 6 h. (g) The percentage of apoptotic cells for each group and time point. ^#^*P* < 0.05 and ^##^*P* < 0.01 versus the H_2_O_2_ group at the same time. Scale bars, 50 *μ*m.

To study the effects of BYHWD on H_2_O_2_-induced apoptosis, cells were stained with PI and Hoechst 33342. The cells that showed strong staining with Hoechst 33342 without PI staining were defined as early apoptotic cells. Similarly, cells showing strong staining with Hoechst 33342 and stained with PI were considered as late apoptotic cells. However, cells stained with Hoechst 33342 and strongly stained with PI were classified as dead. The cells that were stained with Hoechst 33342 and without PI staining were classified as unstressed. The percentage of apoptotic cells was statistically lower (*P* < 0.05 or *P* < 0.01) in groups treated with BYHWD (10, 20, or 30 mg/ml) after both 4 and 6 h of exposure to H_2_O_2_, and the effect was dose-dependent (Figure 6). In summary, these data indicate that BYHWD stops HUVECs from apoptosis induced by H_2_O_2_‒.

### 3.7. Effects of BYHWD on H_2_O_2_-Induced Angiogenesis in HUVECs

**Figure 7:**
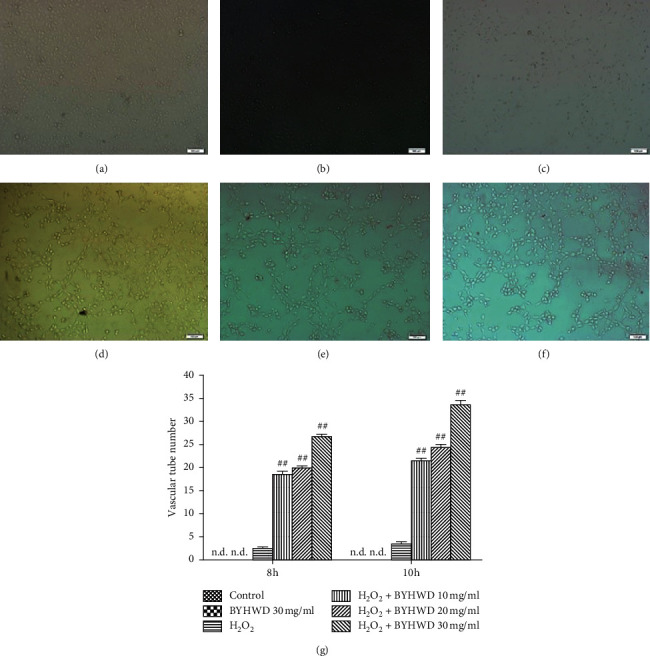
Effects of BYHWD on H_2_O_2_-induced vascular tube formation in HUVECs. (a) Vehicle medium (control) for 10 h. (b) Preincubation with BYHWD (30 mg/ml) for 6 h. (c) H_2_O_2_ (400 *μ*M) for 10 h. (d) Preincubation with BYHWD (10 mg/ml) for 6 h followed by H_2_O_2_ for 10 h. (e) Preincubation with BYHWD (20 mg/ml) for 6 h followed by H_2_O_2_ for 10 h. (f) Preincubation with BYHWD (30 mg/ml) for 6 h followed by H_2_O_2_ for 10 h. (g) Numbers of vascular tubes in HUVECs cultures for each group and time point. ^##^*P* < 0.01 versus the H_2_O_2_ group at the same time. Scale bars, 100 *μ*m.

We measured vascular tube structure as an indicator of H_2_O_2_-induced angiogenesis after treatment with BYHWD. The cells were arranged in stone-like arrangements in the control and BYHWD 30 mg/ml groups, and the cells showed reactive changes, becoming more slender, in the H_2_O_2_-treated group. After different concentrations of BYHWD treatment (10, 20, or 30 mg/ml), the numbers of scattered vascular tube structures were statistically higher compared to the H_2_O_2_ group (*P* < 0.01; Figure 7). These data suggest that BYHWD promotes H_2_O_2_-induced angiogenesis in a dose-dependent manner.

### 3.8. Effects of BYHWD on H_2_O_2_-Induced ROS in HUVECs

**Figure 8:**
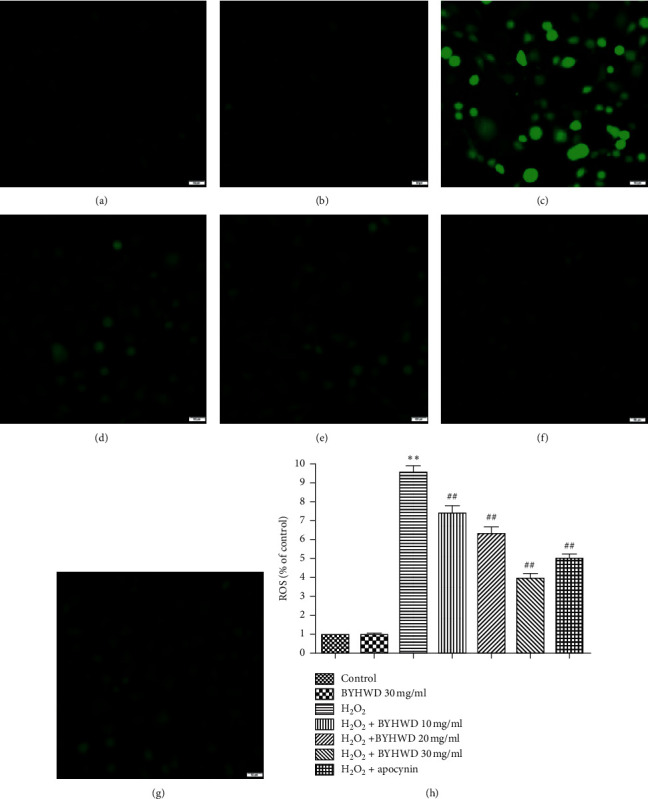
Effects of BYHWD on H_2_O_2_-induced intracellular ROS in HUVECs. Cells were stained with DCFH-DA. (a) Vehicle medium (control) for 6 h. (b) Preincubation with BYHWD (30 mg/ml) for 6 h. (c) H_2_O_2_ (400 *μ*M) for 6 h. (d) Preincubation with BYHWD (10 mg/ml) for 6 h followed by H_2_O_2_ for 6 h. (e) Preincubation with BYHWD (20 mg/ml) for 6 h followed by H_2_O_2_ for 6 h. (f) Preincubation with BYHWD (30 mg/ml) for 6 h followed by H_2_O_2_ for 6 h. (g) Preincubation with apocynin (200 *μ*M) for 1 h followed by H_2_O_2_ for 6 h. (h) Intracellular ROS level in HUVECs for each group. ^*∗∗*^*P* < 0.01 versus the control group. ^##^*P* < 0.01 versus the H_2_O_2_ group. Scale bars, 50 *μ*m.

Cells were stained with DCFH-DA for evaluation of BYHWD effect on H_2_O_2_-induced ROS. Apocynin is an NADPH oxidase inhibitor that inhibits the function of Nox4 and reduces the production of ROS, and it was used as the experimental control for BYHWD treatment. DCFH-produced green fluorescence proves an unusually high ROS level. There were some green fluorescence in control and BYHWD (30 mg/ml) groups and a large amount of green fluorescence in the H_2_O_2_ group. The fluorescence intensity became gradually lower with increasing concentrations of BYHWD treatment in the H_2_O_2_ + BYHWD (10, 20, and 30 mg/ml) groups, compared to the H_2_O_2_ group (Figure 8). The fluorescence intensity was also statistically lower in the H_2_O_2_ + apocynin (200 *μ*M) group to the control group (Figure 8). The results indicate that BYHWD reduces H_2_O_2_-induced ROS synthesis in a dose-dependent manner.

### 3.9. BYHWD Effects on H_2_O_2_-Induced NADPH Oxidase Activity in HUVECs

**Figure 9:**
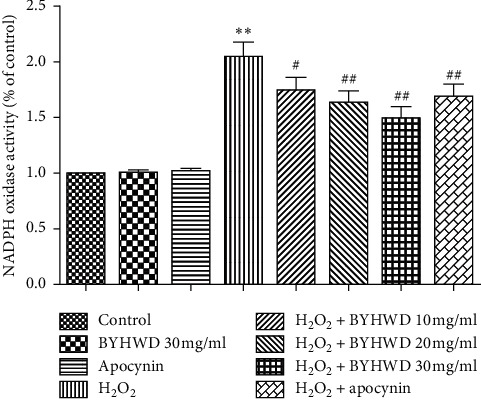
Effects of BYHWD on H_2_O_2_-induced NADPH oxidase activity in HUVECs. NADPH oxidase activity in HUVECs preincubated with BYHWD (10, 20, or 30 mg/ml) for 6 h or apocynin (200 *μ*M) for 1 h followed by H_2_O_2_ for 2 h ^*∗∗*^*P* < 0.01 versus the control group. ^#^*P* < 0.05 and ^##^*P* < 0.01 versus the H_2_O_2_ group.

NADPH oxidase activity in the BYHWD groups was detected through lucigenin chemiluminescence. NADPH oxidase activity was not significantly different among BYHWD (30 mg/ml) or apocynin (200 *μ*M) groups compared to the control group (*P* > 0.05), but the activity of NADPH oxidase was statistically higher in H_2_O_2_ + BYHWD groups (10, 20, and 30 mg/ml), compared to H_2_O_2_ group (*P* < 0.05 and *P* < 0.01). The activity of NADPH oxidase was significantly lower in the H_2_O_2_ + apocynin (200 *μ*M) group compared to the H_2_O_2_ group (*P* < 0.01; Figure 9). The results showed that BYHWD reduces H_2_O_2_-induced NADPH oxidase activity in a dose-dependent manner.

### 3.10. Expression of Nox4 Protein in H_2_O_2_-Induced HUVECs

**Figure 10:**
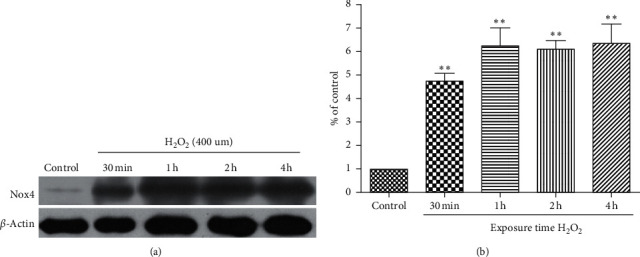
Time course of the effects of H_2_O_2_ on Nox4 protein expression in HUVECs. (a) Western blot of Nox4 protein expression in HUVECs treated with H_2_O_2_ (400 *μ*mol/L) for various lengths of time (30 min or 1, 2, or 4 h). (b) Quantification of Nox4 protein expression in HUVECs for each group. ^*∗∗*^*P* < 0.01 versus the control group.

The time-dependent relationship of Nox4 protein expression in HUVECs treated with H_2_O_2_ for 30 min or 1, 2, or 4 h was evaluated through western blotting. The expression of Nox4 increased from 30 min to 4 h of H_2_O_2_ treatment. Quantification of the data exhibited that the expression of Nox4 was statistically higher compared with the untreated control at all time points (*P* < 0.01 after 30 min exposure to H_2_O_2_; *P* < 0.05 after 1–4 h; Figure 10). These data prove that H_2_O_2_ induces the expression of Nox4 protein in HUVECs in a time-dependent manner.

### 3.11. Effects of BYHWD on H_2_O_2_-Induced Nox4 Protein Expression in HUVECs

**Figure 11:**
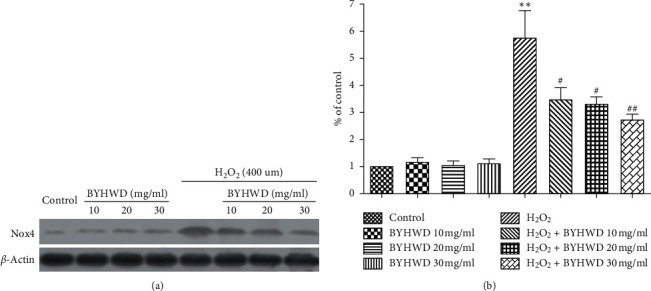
Effects of BYHWD on H_2_O_2_-induced Nox4 protein expression in HUVECs. (a) Western blot of Nox4 protein expression in HUVECs preincubated with BYHWD (10, 20, or 30 mg/ml) for 6 h followed by H_2_O_2_ for 2 h. (b) Quantification of Nox4 protein expression in HUVECs for each group. ^*∗∗*^*P* < 0.01 versus the control group. ^#^*P* < 0.05 and ^##^*P* < 0.01 versus the H_2_O_2_ group.

The western blotting assay was conducted to observe the protein expression of Nox4 in HUVECs treated with BYHWD after the contact with H_2_O_2_ for 2 h. Nox4 expression level was not statistically different among control and BYHWD (10, 20, or 30 mg/ml) groups, but the expression level was significantly higher in the H_2_O_2_-treated group compared to the control group. The expression level of Nox4 was significantly lower in the BYHWD treatment groups (10, 20, and 30 mg/ml) compared with the H_2_O_2_ group (Figure 11). The results showed that BYHWD reduces the expression of Nox4 protein induced by H_2_O_2_ in a dose-dependent manner.

## 4. Discussion

Treatments of acute cerebral ischemic stroke currently recommended by clinical guidelines include intravenous thrombolysis with recombinant tissue plasminogen activator and percutaneous mechanical thrombectomy for large vessel occlusions [28]. However, the complications of acute ischemic stroke include a narrow time window after stroke onset, and the prognosis for secondary CI/R injury, such as fatal intracranial hemorrhage [29]. Therefore, neurorestorative therapies for urgent recanalization of the obstructed artery are likely to be for far longer than those for acute neuroprotection [30, 31]. Previous studies have shown that subacute and chronic recanalization is feasible, safe, and successful for the improvement of cerebral hemodynamics following ischemic stroke [32, 33]. Moreover, our previous research and current studies demonstrate that using drugs to promote blood vessel formation to increase the blood supply in the penumbra area surrounding the ischemic area, i.e., therapeutic angiogenesis, is an alternative strategy for rehabilitating blood circulation, protecting neurons, and improving neurological function after ischemic stroke [17, 34, 35]. VECs sustain vascular integrity and vascular responses to several pathological and physiological processes [36]. Excessive ROS after CI/R has been reported to aggravate endothelial cell injury and prevent angiogenesis [12, 37]. Therefore, protecting VECs against oxidative stress injury could have a therapeutic benefit for cerebral ischemic stroke. Many investigations have shown neuroprotective effects of BYHWD against ischemic stroke and CI/R injury, which involve various mechanisms including inhibiting neural apoptosis and inflammation and nerve cells' differentiation and growth promotion [16, 19]. Our study also showed the protective potential of BYHWD against oxidative stress injury in VECs and angiogenic effects after cerebral ischemic stroke [12]. However, the protective effect and angiogenic mechanisms of BYHWD in VECs after ischemic stroke are currently unknown. In an attempt to understand this mechanism, we evaluated BYHWD effects on angiogenesis and neuroprotection after CI/R injury in rats and the underlying angiogenic effects in an H_2_O_2_-induced oxidative stress model in HUVECs.

This study demonstrated that BYHWD significantly improves neurological deficits and spatial learning and memory and significantly increases CBF and MVD in the ischemic area after CI/R injury in rats. Moreover, BYHWD also decreases intracellular ROS and Nox4 protein expression, inhibits cell apoptosis, and induces vascular tube formation in H_2_O_2_-treated HUVECs. These data support the idea that BYHWD promotes neurological function recovery through increasing angiogenesis by downregulation of the Nox4/ROS pathway in VECs after ischemic stroke.

There are three different types of neurovascular remodeling in the ischemic area after acute cerebral ischemia in adults: vasculogenesis, angiogenesis, and arteriogenesis. At present, angiogenesis is the best-understood mechanism of neurovascular remodeling after cerebral ischemia [38], which includes the process of proliferation and migration from the activated endothelial cells, and then branching, remodeling, and lumen formation from the existing vascular collateral to expand and extend into a new capillary network. Angiogenesis is an essential mechanism for neurovascular remodeling because it helps in enhancing cerebral blood flow and glucose metabolism in the ischemic boundary zone in the late cerebral ischemic stroke stages [30, 39]. Thus, finding strategies for promoting angiogenesis in ischemic stroke may lead to the development of potential therapeutic targets. MVD is regarded as a valuable parameter for quantitatively evaluating angiogenesis after ischemic stroke [40]. Factor VIII antigens have been used for this purpose because they stain both preexisting mature vessels and newly formed blood vessels as well [27]. In this study, MVD was examined by immunofluorescence staining of factor VIII antigens. MVD increased significantly after CI/R, and BYHWD further upregulated MVD at 4 w after CI/R. Moreover, we quantified CBF and assessed neurovascular remodeling for functional blood supply. Our results also show that BYHWD improved the CBF in the ischemic area after cerebral infarction in a time-dependent manner. Meanwhile, we measured cell viability, apoptosis, and vascular tube formation in an H_2_O_2_-induced HUVECs injury model. Our results also indicate that BYHWD increases cell viability, inhibits apoptosis, and induces vascular tube formation in a dose-dependent manner in this model. Therefore, our results demonstrate that BYHWD activates angiogenic effects in vivo and in vitro and may eventually increase local blood supply to promote neurological function recovery after cerebral infarction.

NADPH oxidase derived ROS are involved in endothelial permeability and dysfunction, vascular remodeling, cell growth and migration, inflammation, apoptosis, and senescence, which contribute to atherosclerosis, diabetes, hypertension, and acute lung sepsis/injury [41]. Nox4 is a member of the Nox family of ROS-producing NADPH oxidases in vascular endothelial cells [42, 43]. Ischemia/reperfusion injury induces upregulation of Nox4, which, as a key source of oxidative stress, offers a new therapeutic target in cerebrovascular disease [12, 44]. HUVECs are the predominantly used model for ischemic cardiocerebrovascular disease research [45, 46]. We used H_2_O_2_-induced oxidative stress injury in HUVECs as a model of CI/R. Our results demonstrate that BYHWD inhibits H_2_O_2_-induced cell apoptosis and increases the cell viability loss induced by H_2_O_2_ along with its increasing concentration. BYHWD reduces H_2_O_2_-induced NADPH oxidase activity and Nox4 protein expression in HUVECs in a concentration-dependent manner. Moreover, BYHWD reduces H_2_O_2_-induced ROS production. In contrast, apocynin, a specific inhibitor of NADPH oxidase, inhibits H_2_O_2_-induced ROS production and NADPH oxidase activity. Thus, BYHWD exerts angiogenic effects through the downregulation of Nox4, which in turn results in the dropping of ROS production.

## 5. Conclusion

The current study indicates that BYHWD improves neurological deficits and spatial learning and memory and increases MVD and CBF in the ischemic area after CI/R injury. BYHWD also increases cell viability, inhibits apoptosis, induces vascular tube formation, decreases intracellular ROS generation, and reduces Nox activity and Nox4 protein expression in H_2_O_2_-induced oxidative stress injury in HUVECs. Therefore, BYHWD exerts angiogenic effects against cerebral ischemic injury through the downregulation of Nox4, which results in the reduction of ROS generation, which may be a potential therapeutic strategy for cerebral ischemic stroke.

## Data Availability

The dataset supporting the conclusions of this study are publicly available.
